# Dupilumab modulates specific IgE mite responses at the molecular level in severe T2-high atopic dermatitis: A real-world experience

**DOI:** 10.3389/fmed.2022.939598

**Published:** 2022-08-12

**Authors:** Ruperto González-Pérez, Paloma Poza-Guedes, Elena Mederos-Luis, Inmaculada Sánchez-Machín

**Affiliations:** ^1^Allergy Department, Hospital Universitario de Canarias, Santa Cruz de Tenerife, Spain; ^2^Severe Asthma Unit, Hospital Universitario de Canarias, Santa Cruz de Tenerife, Spain

**Keywords:** atopic dermatitis, dupilumab, specific IgE, mites, personalized allergy molecular diagnosis

## Abstract

**Background:**

Atopic dermatitis (AD) is regarded as a chronic systemic disease which is characterized by a robust overexpression of type 2 related cytokines, with increased total IgE levels and a concomitant sensitization to common allergens. Dupilumab, a fully human monoclonal antibody (mAb) to IL-4Rα that inhibits both IL-4 and IL-13 signaling, has previously shown a marked and rapid improvement when treating the moderate-to-severe forms of AD. We sought to evaluate the real-world evidence (RWE) of dupilumab in the modulation of total and specific IgE (sIgE) serum levels to a panel of molecular house dust mites (HDM) and storage mites (SM) allergens in patients with severe AD.

**Methods:**

Demographic and clinical data for severe AD adult patients receiving dupilumab treatment (300 mg every 2 weeks) were reviewed. Mean (standard deviations SD) values and percent changes from baseline in total and sIgE to the complete HDM and SM extracts, and 14 individual molecular allergens were measured over 52 weeks.

**Results:**

Significant (*p* < 0.05) changes in mean total IgE levels were observed from baseline to week-52 after treatment with dupilumab. Despite no changes were found in sIgE against the extract of HDM during the 52-week treatment with dupilumab, baseline mean levels from 7 out of 14 individual molecular mite allergens -Der p 1, Der p 2, Der p 5, Der p 7, Der p 21, Der p 23, and Lep d 2- were significantly (*p* < 0.05) decreased—after 52 weeks of treatment with dupilumab.

**Conclusions:**

Dupilumab therapy for 52 weeks resulted in a profound reduction in blood levels of total IgE and allergen-specific IgE to both HDM and SM at the molecular level in adults with severe AD under RWE conditions. The potential benefits of these concomitant immunomodulatory effects after treatment with dupilumab should be explored to a greater extent.

## Introduction

Atopic dermatitis (AD), an inflammatory, chronically relapsing and intensely pruritic skin disease, is nowdays considered a systemic Type 2 inflammation driven disease, enclosing a specific CD4+ T helper type (Th) 2 cell response and the activation of several T-cell lineages, such as Th1, Th17/interleukin (IL)-23, and Th22 (1, 2). Former clinical and experimental studies indicate that house dust mites (HDM) can play a critical role in the pathogenesis of AD in sensitized and genetically predisposed subjects (3, 4). The introduction of Precision Allergy Molecular Diagnosis (PAMD@) has a major effect on analytic specificity and allergy diagnosis, to a comprehensive assessment of the patient's specific IgE (sIgE) binding to a panel of individual allergens (5).

Dupilumab, the first fully human monoclonal IgG4 antibody inhibiting the signaling of IL-4 and IL-13, significantly reduces type 2 biomarkers in circulating serum and lesional skin (6, 7). Nowadays, changes in allergen-specific IgE serum levels have only been described for allergen extracts in subjects with moderate-to-severe AD from clinical trials with dupilumab (8, 9). Herein, we aimed to investigate the real-world evidence (RWE) effects of after 52 weeks of treatment with dupilumab in total and mite-molecular IgE serum levels in a cohort of individuals afflicted with the severe extrinsic AD phenotype subjected to a high local environmental mite exposure (10, 11).

## Materials and methods

### Subjects

We consecutively recruited patients from May 2020 to November 2021 from the Outpatient Allergy Clinic & Severe Asthma Unit at Hospital Universitario de Canarias (Tenerife, Spain). Eligibility criteria included subjects in treatment with dupilumab 300 mg every 2 weeks (300 mg-q2w) for 52 weeks with a clinician confirmed diagnosis of severe AD with the T2-high endotype (extrinsic subtype) according to current guidelines (12).

Clinical data -including Scoring Atopic Dermatitis (SCORAD), Eczema Area and Severity Index (EASI) score, Investigator's Global Assessment (IGA) and peak pruritus numerical rating scale (PNRS)- were retrieved from the patients' medical records 4 weeks before treatment with dupilumab 300 mg-q2w (Time 0, T0), 26 weeks after starting therapy with dupilumab (Time 1, T1), 32 weeks after dupilumab (Time 2, T2) and finally after completing 52 weeks with dupilumab (Time 3, T3). The study was conducted according to the guidelines of the Declaration of Helsinki and approved by the Institutional Ethics Committee of CEIC Hospital Universitario de Canarias, Tenerife, Spain with the reference number P.I.-2017/72 on 30 October 2017.

Pregnant and breast-feeding women and patients receiving allergen immunotherapy (AIT), and/or other biologics apart from dupilumab were excluded from the investigation. Blood specimens were collected for all subjects, identified with a code label, stored at −40°C and immediately thawed in preparation for *in vitro* analysis to evaluate median values and percent changes from baseline in total and allergen-specific IgE over the 52 weeks follow up.

### Skin prick test and mite allergenic extracts

Percutaneous tests were performed according to European standards (13) with standardized allergenic extracts of *Dermatophagoides pteronyssinus, Blomia tropicalis, Lepidoglyphus destructor*, and *Tyrophagus putrescentiae* (Diater, Madrid, Spain). Saline (0.9%) and histamine (10 mg/ml) were respectively included as negative and positive controls. Antihistamines were drop back seven days in advance to each SPT, with wheal diameters >3 mm considered positive after a 20 min immediate reading.

### Serological workup

Total IgE levels, sIgE to the whole *D. pteronyssinus* and *B. tropicalis* extract, and 14 individual molecular allergens -Der p 1, Der p 2, Der p 5, Der p 7, Der p 10, Der p 11, Der p 20, Der p 21, Der p 23, Blo t 5, Blo t 10, Blo t 21, Lep d 2, and Tyr p 2- were measured by a MedTech company (MacroArray Diagnostics, Viena, Austria), according to the manufacturer's instructions. In brief, the Allergy Explorer (ALEX^®^) test is a multiplex array containing 282 reagents (157 extractive allergens and 125 molecular components). The different allergens and components are coupled onto polystyrene nano-beads, and then the allergen beads are deposited on a nitrocellulose membrane, as previously published (14). Total IgE levels were expressed in international units per unit volume (IU/mL), sIgE levels were expressed in kU_A_/L. Values ≥0.35 kU_A_/L were regarded as positive.

### Statistical data

Demographic features were summarized by means and standard deviations for continuous variables and percentages for categorical variables. To compare differences analysis of variance, Kruskal–Wallis, Mann–Whitney U and Chi-square tests are required for parametric continuous, non-parametric continuous, and categorical variables respectively. A *P*-value of <0.05 was considered statistically significant. All statistical data were analyzed using GraphPad Prism version 8.0.0 for Windows, GraphPad Software, La Jolla, California, USA.

## Results

### Demographic characteristics of patients

We finally selected 12 (out of 22) European-American ethnicity subjects from the outpatient allergy office –*10 males and 2 females, median age 27.0 (19–57) years of age*- who met the inclusion criteria and a previous clinician-confirmed diagnosis of AD ongoing for more than 15 years. Considering SCORAD >40 and EASI >21 as markers for the severe forms of (15), all selected subjects showed upon inclusion a median SCORAD and EASI of 84.5 (54.0–96.4) and 62.40 (27.0–70.0), respectively. A median Investigator's Global Assessment (IGA) score of 4 (severe on a scale of 0–4) and a median peak pruritus numerical rating scale (NRS) of 8 (severe on a scale of 0–10) was also quantified for all subjects at baseline.

More than 80% (10 out 12 subjects) had a former family history of atopy and regarding comorbidities, 91.66% patients were afflicted with allergic rhinitis and/or asthma, and 25.0% had a clinically confirmed food allergy (milk, seafood and/or tree nuts) associated (Table 1).

**Table 1:** Descriptive statistics at baseline.

**Study population (*N* = 12)**	**Severe atopic dermatitis**
Age (y.o.) median (range)	27.0 (19–57)
Sex (M/F)	(10/2)
SCORAD index >40 *N* (%)	12 (100)
EASI score >21 *N* (%)	12 (100)
IGA = 4 *N* (%)	12 (100)
PNRS median (range)	8 (7–9)
Systemic cyclosporin/steroids *N* (%)	12 (100)
Rhinitis and/or Asthma *N* (%)	11 (91.66)
Food Allergy *N* (%)	3 (25.0)
Family History of Atopy *N* (%)	10 (83.3)
Total IgE (UI/ml) median (range)	4,246 (221–19,370)
sIgE *D. pteronyssinus* (kU/L) > 100 *N* (%)	12 (100)
sIgE *B. tropicalis* (kU/L) median (range)	64 (8.11–>100)
Blood Eosinophils/mm^3^ median (range)	390 (40–1,800)

SCORAD, Scoring Atopic Dermatitis; EASI, Eczema Area and Severity Index; IGA, Investigator's Global Assessment; PNRS, Pruritus Numerical Rating Scale D. pteronyssinus: Dermatophagoides pteronyssinus. B. tropicalis: Blomia tropicalis. Median values are shown.

Concerning the need of systemic medication for AD at baseline, patients were in treatment with either cyclosporin (75% of subjects with a median dose of 75 mg/day), steroids (16.6% with a median dose of prednisone 15 mg/day) or azathioprine (50 mg/day in 1 individual).

### Quantification of basal total IgE, SPT, and SIgE to the extract of *D. pteronyssinus* and *Blomia tropicalis* and mite molecular profile

All patients confirming their eligibility for the study showed a marked Th2-high endotype -featuring elevated peripheral eosinophil count, total IgE and aeroallergen-specific IgE levels. Basal total IgE (before treatment with dupilumab) ranged from 221.0 to 19.370.0 IU/mL, with a median value of 3765.0 IU/mL. Basal median blood eosinophils showed a value of 390 (40–1,800) eosinophils/μL. All enrolled individuals had a positive SPT and serum sIgE (≥0.35 kU/l) against both *D. pteronyssinus* and *B. tropicalis* (crude extract), with median values of >100 and 64 (8.11 to>100) kU/L, respectively (Table 1).

Globally, all patients were simultaneously sensitized to 3 or more of the 14 mite allergens included in the customized molecular panel. Regarding the frequency of sIgE-binding to individual molecular allergens, Der p 2, Der p 21, and Der p 23 led the sIgE immunoresponse in all individuals (100%), followed by Der p 1 and Der p 5 (83.33%) subjects, while 75.0% individuals were sensitized to Der p 7. Minor allergens as Der p 10 were only present in 8.33%, while Der p 11 and Der p 20 were found in 16.66% of the investigated serum samples. Considering storage mites, Lep d 2 (58.8%) and Blo t 5 (50.0%) were the most frequently identified allergens in the current cohort.

In relation to the aggregation of allergens, despite the repertoire of IgE-recognized molecules was highly polyclonal, a preponderant specific pattern could not be found (Table 2). The quantitative basal mean values of sIgE (kU/L) were the highest for Der p 2 (52.64 ± 5.28), followed by Der p 23 (45.0 ± 12.06), Der p 21 (43.71 ± 22.79), Der p 5 (42.25 ± 16.64), Der p 1 (24.6 ± 17.87), Blo t5 22. 32 ± 13.62 Der p 7 (20.05 ± 20.44), and Lep d 2 (13.95 ± 11.56).

**Table 2:** Specific IgE profiles to 14 allergen molecules from *Dermatophagoides pteronyssinus, Blomia tropicalis, Lepidoglyphus destructor*, and *Tyrophagus putrescentiae* in subjects afflicted with severe atopic dermatitis tested with microarray.

***n* = 12**	**Number of molecules**	**Der p 1**	**Der p 2**	**Der p 5**	**Der p 7**	**Der p 10**	**Der p 11**	**Der p 20**	**Der p 21**	**Der p 23**	**Blo t 5**	**Blo t 10**	**Blo t 21**	**Lep d 2**	**Tyr p 2**
1	3	*	*							*					
1	5		*		*				*	*				*	
1	6	*	*	*					*	*	*				
2	7	*	*	*	*				*	*				*	
2	8	*	*	*	*				*	*	*			*	
1	8		*	*					*	*	*		*	*	*
1	10	*	*	*		*			*	*	*	*	*	*	
1	10	*	*	*	*				*	*	*		*	*	*
2	12	*	*	*	*		*	*	*	*	*		*	*	*

Profiles are ordered by the increasing number of recognized molecules.

Asterisk (^*^) indicates specific IgE sensitization to single molecular allergens.

### Evolution of clinical severity and pruritus after therapy with dupilumab 300 mg-Q2w for 52 weeks

A significant improvement in both SCORAD index and EASI score was reported at weeks 26 and 52 in all subjects compared to their baseline scores (Table 3). In addition, at weeks 26 and 52, 83.33% patients receiving dupilumab had an IGA score of 0 or 1 and an improvement of 2 points or more on the IGA from the baseline score. Also, at weeks 26 and 52, a reduction of at least 5 points in the peak score on the PNRS was found in all patients after treatment with dupilumab in contrast to their baseline scores (*P* < 0.05 for all comparisons). Concomitant systemic medication was discontinued (ciclosporin, steroids and/or azathioprine) in all subjects at week 52 after treatment with dupilumab.

**Table 3:** Scoring features and routine laboratory testing in patients (*n* = 12) with severe atopic dermatitis at baseline and follow-up after 26 and 52 weeks of treatment with dupilumab 300 mg every 2 weeks for 52 weeks.

	**Baseline**	**Week 26**	**Week 52**
SCORAD index	74.93 ± 15.89	25.12 ± 12.75*	15.07 ± 7.97*
EASI score	50.75 ± 17.83	5.94 ± 2.68*	2.24 ± 2.17*
IGA	4 ± 0.0	1.33 ± 0.7*	1.0 ± 0.81*
PNRS	7.91 ± 0.66	1.75 ± 1.38*	1.26 ± 0.76*
BSA (%)	70.69 ± 16.07	14.0 ± 7.19*	11.9 ± 8.65*
POEM score	24.79 ± 3.41	13.24 ± 4.68*	9.39 ± 5.23*
Eosinophils/mm^3^ (blood)	562.5 ± 563.47	1,113.42 ± 1,005.94	778.12 ± 691.16
sIgE *D. pteronyssinus* (kU/L)	100 ± 0.0	100 ± 0.0	100 ± 0.0
sIgE *B. tropicalis* (kU/L)	75.02 ± 39.61	64.0 ± 29.48	19.92 ± 26.03*
Serum Total IgE (UI/mL)	6,751.88 ± 7,626.39	3,040.5 ± 2,546.61*	3,149.33 ± 2,513.24*

Mean values and standard deviations are shown.

Asterisk (^*^) indicates statistical significance (p < 0.05). D. pteronyssinus: Dermatophagoides pteronyssinus. B. tropicalis: Blomia tropicalis.

### Total serum IgE and mite molecular responses after treatment with dupilumab 300 mg every 2 weeks for 52 weeks

Significant (*p* < 0.05) changes in mean total IgE levels were observed from baseline (6,751.88 ± 7,626.39 IU/mL) to week-26 (3,040.5 ± 2,546.61 IU/mL) and week-52 (3,149.33 ± 2,513.24 IU/mL) after treatment with dupilumab. No changes were found in sIgE against the extract of *D. pteronyssinus*-as mean values remained >100 kU/L- during the 52-week treatment with dupilumab. Mean sIgE levels against the extract of *B. tropicalis* were significantly reduced from baseline (75.02 ± 39.61 kU/L) to (19.92 ± 26.03 kU/L) by week 52.

**Figure 1:**
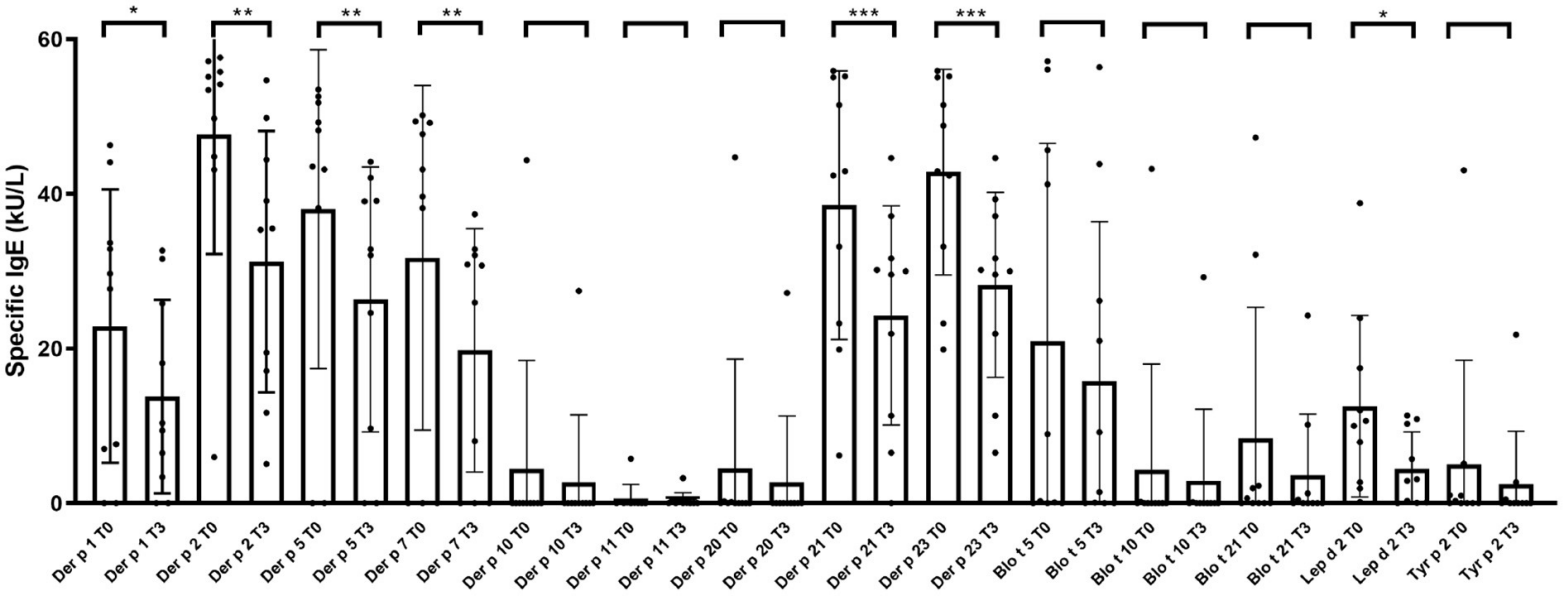
Evolution of serum specific IgE profiles to 14 allergen molecules from *Dermatophagoides pteronyssinus, Blomia tropicalis, Lepidoglyphus destructor*, and *Tyrophagus putrescentiae* in subjects afflicted with severe atopic dermatitis tested with microarray, before (T0) and after treatment with dupilumab 300 mg every 2 weeks for 52 weeks (T3). Asterisk indicate statistical significance (*p < 0.05; **P < 0.01; and ***p < 0.005).

Interestingly, baseline mean levels (kU/L) from 7 out of 14 (50%) individual molecular mite allergens -Der p 1 (24.6 ± 17.87), Der p 2 (52.64 ± 5.28), Der p 5 (42.25 ± 16.64), Der p 7 (20.05 ± 20.44), Der p 21 (43.71 ± 22.79), Der p 23 (45.0 ± 12.06) and Lep d 2 (13.95 ± 11.56)- were significantly (*p* < 0.05) decreased -Der p 1 (11.82 ± 11.5), Der p 2 (28.63 ± 15.64), Der p 5 (25.74 ± 18.02), Der p 7 (14.78 ± 16.08), Der p 21 (29.31 ± 23.31), Der p 23 (27.0 ± 11.99), and Lep d 2 (3.72 ± 4.35)- after 52 weeks of treatment with dupilumab. In addition, despite no statistical significance was reached, even a decreased in the mean sIgE levels was confirmed for Der p 10, Der p 11 and Der p 20 (regarded as minor allergens from *D. pteronyssinus*), and also for the major allergens (Blo t 5 and Blo t 21) from *B. tropicalis* and *T. putrescentiae* (Tyr p 2), respectively, after 52 weeks of treatment with dupilumab (Figure 1).

### Safety and adverse events

Two subjects (16.66%) had adverse events developing mild conjunctivitis after 16-weeks of treatment with dupilumab. Conjunctivitis was controlled after using moisturizing sodium hyaluronate eye drops. None of the patients had to discontinue therapy with dupilumab during the study period.

## Discussion

The identification of *in vitro* biomarkers -including the reduction of sIgE- and their direct association with disease severity and the potential prediction of severe disease are of great clinical interest in monitoring the effects of targeted therapy for different allergic conditions (16–20).

Previous studies provide evidence that AD and allergic asthma might share related drivers -i.e., IL-4 and IL-13- and that both conditions may benefit from a common therapeutic inhibition of these Th2 cytokines with dupilumab (21).

The contributing role of mites and a high prevailing capacity for sIgE sensitization -based on the frequency of IgE-binding- has been previously described in the pathogenesis and severity of AD in populations from diverse geographical areas and ethnicities (22, 23). In the present investigation, and in addition to the supporting clinical outcomes after therapy with dupilumab, a significant immunomodulation in the sIgE molecular profile to major and mid-tier mite allergens has been found in a RWE subset of patients afflicted with severe AD. The current discernment of B cell activation and differentiation during development and later persistence or resolution of human IgE-mediated allergic diseases is still limited. The lifespan of IgE plasma cells and the conditions in which new IgE plasma cells are generated are essential questions in the recognition of potential pathways for therapeutic intervention in allergic disease (24). In respect to perennial allergens (i.e., mites), persistent IgE could be attributed to long-lived IgE plasma cells or short-lived IgE plasma cells continuously generated from a memory reservoir. As plasmablasts originate from non-IgE memory cells, it has been speculated that dupilumab resulted in a marked reduction of serum allergen-specific IgE -an IL-4 and IL-13-dependent process- as a large portion of circulating IgE in AD patients derives from short-lived IgE plasma cells elicited in recent class-switching events (25).

The disease-modifying effects of AIT are also associated with immune modulation of the innate and adaptive immune responses. In fact, successful AIT has been related to the induction of allergen-specific blocking IgG antibodies, inhibiting allergen-induced mast cell and basophil degranulation and IgE-facilitated allergen presentation to T cells (26–28). In addition to AIT, it has been shown that circulating IgE can be effectively reduced by the binding of omalizumab –a recombinant humanized monoclonal anti-IgE-antibody– to the binding site of the high affinity FcεRI receptor on the IgE antibody priming the downregulation of the FcεRI receptor on basophils and mast cells (29). Interestingly, despite both AIT and omalizumab have confirmed a clinical benefit in a variety of IgE-mediated allergic diseases -i.e., allergic rhinitis and/or asthma and chronic spontaneous urticaria- their role in AD is still controversial (30–32). In the present report, the significant reduction in the molecular sIgE to mite allergens may be only speculated as a part in the mechanistic puzzle to a favored outcome after dupilumab therapy in severe AD individuals. Furthermore, although no changes in the *in vitro* sIgE to the extract of *D. pteronyssinus* were identified -as median sIgE values remained >100 kU/L during the 52 weeks of treatment with dupilumab-, PAMD@ unveiled a significant decrease in major and mid-tier molecular allergens from *D. pteronyssinus*. These observations would suggest that only sIgE antibody responses to the serodominant -but not those molecules with the lowest prevalence- are being continually stimulated by Th2 dependent immune responses. The current research has potential bias as the study was performed in a single center, with a limited number of subjects, and data from T2 could not be retrieved in 5 subjects due to local COVID-19 pandemic restrictions. Also, the identification of minor molecules as Der p 18, Der f 13, Der f 14, Der f 32, and Der f Alt a 10, regarded as mite immunologic markers for AD, was neither assessed in the intended population (33, 34).

Despite previous trials with dupilumab treatment resulted in a prompt decrease of total and allergen serum sIgE in moderate-to-severe AD (35), this is to our knowledge the first RWE report to confirm a significant and sustained reduction in blood levels of mite sIgE at the molecular level after 52 weeks of treatment with dupilumab in a selected cohort of adult patients with severe AD. In line with recent reports, (36–38), our data suggest that a long-term clinical benefit could be sustained even after discontinuing dupilumab therapy in adult patients with AD, similar to the sustained decreases in serum sIgE levels and long-term clinical improvements observed in subjects with allergic rhinitis and/or asthma during and after AIT. Either these results may extend the potential benefit of dupilumab, not only from severe AD to other coexisting atopic conditions but also in the so-called atopic march, it is an issue that certainly deserves further research.

## Data availability statement

The raw data supporting the conclusions of this article will be made available by the authors, without undue reservation.

## Ethics statement

The studies involving human participants were reviewed and approved by Institutional Ethics Committee of CEIC Hospital Universitario de Canarias, Tenerife, Spain with the reference number P.I.-2017/72 on October 30, 2017. The Ethics Committee waived the requirement of written informed consent for participation.

## Author contributions

RG-P and PP-G: conceptualization, data curation, writing—original draft preparation, and funding acquisition. RG-P, PP-G, and EM-L: methodology. PP-G: software. PP-G and IS-M: validation and formal analysis. IS-M, RG-P, PP-G, and EM-L: investigation and writing—review and editing. IS-M: resources. RG-P, PP-G, and IS-M: project administration. All authors have read and agreed to the published version of the manuscript.

## Funding

This research was funded by Fundación Canaria Instituto de Investigación Sanitaria de Canarias (FIISC), Servicio Canario de Salud, grant number OA17/042.

## Conflict of interest

The authors declare that the research was conducted in the absence of any commercial or financial relationships that could be construed as a potential conflict of interest.

## Publisher's note

All claims expressed in this article are solely those of the authors and do not necessarily represent those of their affiliated organizations, or those of the publisher, the editors and the reviewers. Any product that may be evaluated in this article, or claim that may be made by its manufacturer, is not guaranteed or endorsed by the publisher.

## Abbreviations

AD: Atopic Dermatitis
HDM: House Dust Mite
PAMD@: Personalized Allergy Molecular Diagnosis
*D. pteronyssinus*: *Dermatophagoides pteronyssinus*
*B. tropicalis*: *Blomia tropicalis*
SPT: Skin Prick Test
RWE: Real World Evidence.
